# Asiatic Cholera

**Published:** 1883-12

**Authors:** 


*Asiatic Cholera.* A Report of an outbreak of Epidemic Cholera in 1876 at a Camp near Murree. By CHARLES MOORE JESSOP, M.R.C.P., Brigade Surgeon H.M.'s British Forces. London: H. K. Lewis. 1883.

This brief outline of the author's personal experience of a disease which is now attracting so much notice, both from the public and from scientific investigators, directs attention to one special point in the etiology of cholera. He endeavours to show that the disease, as it is seen in epidemic form, is a "selective" disease, seizing upon those who have some invisible and unhealthy defect, and hence attention to the minor conditions of health known as dyspepsia is imperative in "cholera years." He attributes much of the cholera he saw to the "plum dough and boiled cows' heel messes," in which the troops generally indulged.

